# A153 IL10-PRODUCING GUT-RESIDENT TR1 CELLS ARE FOUND AT HIGH FREQUENCIES IN HEALTHY HUMANS AND MICE AND MAY PROTECT AGAINST MURINE ACUTE COLITIS

**DOI:** 10.1093/jcag/gwae059.153

**Published:** 2025-02-10

**Authors:** C Poloni, A Sze, S Lim, M Esmaeilzadeh, X Wang, L Cook, T Steiner

**Affiliations:** The University of British Columbia, Vancouver, BC, Canada; The University of British Columbia, Vancouver, BC, Canada; The University of British Columbia, Vancouver, BC, Canada; The University of British Columbia, Vancouver, BC, Canada; The University of British Columbia, Vancouver, BC, Canada; The University of Melbourne, Melbourne, Victoria, Australia; The University of British Columbia, Vancouver, BC, Canada

## Abstract

**Background:**

Inflammatory bowel disease (IBD) affects an estimated 270,000 people in Canada and is increasing in prevalence. Current treatments, such as anti-TNFα mAbs, block inflammatory pathways, but require repeated dosing and do not reverse intestinal fibrosis. Additionally, a significant subset of IBD patients have relapsing disease and do not respond to biologics. We have shown type 1 regulatory cells (Tr1s) are capable of suppressing inflammation via IL-10, promote gut-healing and barrier function via IL-22, and are long-lasting in mouse models.

**Aims:**

We aimed to evaluate adoptive transfer of Tr1 cells as a therapy for IBD. We hypothesized that Tr1 cells can prevent inflammatory damage and fibrosis in the context of IBD.

**Methods:**

The frequency of non-FOXP3 (Tr1) regulatory cells were analyzed from IL-10-eGFP mice and human gut samples from the Australian Donation and Transplantation Biobank to determine the major source of gut-resident T-cell-derived IL-10. Adoptive transfer experiments were performed using 9-10 week old C57BL/6 mice, where mice were briefly irradiated, injected with 1x10^6^ FOXP3^neg^ IL-10^+^ (Tr1) cells or PBS prior to administration of dextran sulfate sodium (DSS) to induce acute colitis. Weight and disease score were measured for 14 days, followed by histologic analysis of gut tissue and flow cytometric analysis of adoptively transferred (Thy1.1^+^) and endogenous (Thy1.2^+^) T cells.

**Results:**

Tr1 cells act as the major source of intestinal CD4^+^ gut-resident derived IL-10, as compared to FOXP3^+^ T regulatory cells in mice and humans (p=0.0051, 0.0156). Further, mice receiving Tr1 adoptive transfer therapy have decreased weight loss at day 7 post DSS administration (p = 0.0163), increased colon length (p=0.0406), decreased colon thickening (p=0.0234), and decreased blinded histologic scoring (p=0.0229) at experimental endpoint. Furthermore, successfully engrafted adoptively transferred Tr1 cells have increased expression of T regulatory marker ICOS in the blood, spleen, and mesenteric lymph node of mice receiving cellular therapy, as compared to endogenous CD4^+^ T cells (p=0.0003, 0.003, <0.0001). Preliminary experiments suggest human CD4^+^ ICOS^+^ T cells secrete high levels of IL-10.

**Conclusions:**

Tr1 cells are the major T cell producers of anti-inflammatory cytokine IL-10 in both human and murine intestine, producing more IL-10 than FOXP3^+^ regulatory T cells. Adoptive transfer of Tr1 cells into a murine model of acute colitis protects against weight loss and colon remodeling. Further, these cells are long-lived and detectable up to 8 weeks post transfer. Together, this data suggests Tr1 cellular therapy may be a promising treatment for IBD.

**Funding Agencies:**

CIHR

